# ZnO NRs/rGO Photocatalyst in a Polymer-Based Microfluidic Platform

**DOI:** 10.3390/polym15071749

**Published:** 2023-03-31

**Authors:** Aini Ayunni Mohd Raub, Ida Hamidah, Asep Bayu Dani Nandiyanto, Jaenudin Ridwan, Mohd Ambri Mohamed, Muhamad Ramdzan Buyong, Jumril Yunas

**Affiliations:** 1 Institute of Microengineering and Nanoelectronics (IMEN), Universiti Kebangsaan Malaysia (UKM), Bangi 43600, Malaysia; 2 Faculty of Engineering Education, Universitas Pendidikan Indonesia, Jl. Dr. Setiabudhi 207, Bandung 40154, Indonesia

**Keywords:** microfluidic reactor, SU-8 master mold, reduced graphene oxide/zinc oxide nanorods, methylene blue, water treatment

## Abstract

This paper reports the development of ZnO NRs/rGO-based photocatalysts integrated into a tree-branched polymer-based microfluidic reactor for efficient photodegradation of water contaminants. The reactor system includes a photocatalytic reactor, tree-branched microfluidic channels, and ZnO nanorods (NRs) coated with reduced graphene oxide (rGO) on a glass substrate within an area of 0.6 × 0.6 cm^2^. The ZnO NRs/rGO acts as a photocatalyst layer grown hydrothermally and then spray-coated with rGO. The microfluidic system is made of PDMS and fabricated using soft lithography (micro molding using SU-8 master mold patterned on a silicon wafer). The device geometry is designed using AutoCAD software and the flow properties of the microfluidics are simulated using COMSOL Multiphysics. The microfluidic platform’s photocatalytic process aims to bring the nanostructured photocatalyst into very close proximity to the water flow channel, reducing the interaction time and providing effective purification performance. Our functionality test showed that a degradation efficiency of 23.12 %, within the effective residence time of less than 3 s was obtained.

## 1. Introduction

Water treatment is an essential part of the water cycle that must be managed throughout the water management cycle: from freshwater intake, treatment, distribution, use, collection, and post-treatment to reuse and eventually return to the environment from which the water source was recovered. Compared to water supply concerns, water management receives less societal and political attention, especially in relation to water scarcity. According to the United World Water Development Report 2017, better wastewater management leads to social, environmental, and economic advantages critical for long-term development and achieving the 2030 Agenda for Sustainable Development [1]. Moreover, due to the volume and cost of storing drinkable water on the international space station (ISS), recycling used water is the best option for obtaining clean water. The Water Processor Assembly (WPA) is the technology currently in use on the ISS, and it uses high temperature and pressure to complete an oxidation catalytic reaction for the elimination of volatile organic compounds [2]. The problem with this technology is that its high operating temperature and pressure necessitate frequent replacement of its weak parts [3]. A novel photocatalytic water purification microreactor that operates at standard pressure and temperature must be designed to overcome these difficulties.

Numerous metal oxides have been used as photocatalysts in water treatment. By implementing binary or ternary semiconductors, band gap narrowing can be achieved for a wide range of optoelectronics applications [4,5]. A ZnO and GO-based nanocomposite has successfully degraded different dyes such as MB (methylene blue) [6,7], MO (methyl orange) [8], CV (crystal violet) [9], RhB (rhodamine B) [10]; removed heavy metals [11]; and performed bacterial disinfection [12,13]. ZnO nanorods and nanowires have been investigated extensively among 1D nanostructures due to their ease of nanomaterial fabrication and device applications [14]. Because of their greater surface-to-volume ratio, ZnO nanorods and nanowires have superior organic dye adsorption and photodegradation efficiency than nanoparticles. Despite this, ZnO’s photocatalytic effectiveness is limited since it cannot be sustained at higher pH, rapidly decays in the second run, and has a high rate of electron–hole recombination and photo-corrosion [15,16]. Because of their simplicity, the accessibility of equipment, low cost, and predictable growth temperatures, hydrothermal methods utilizing spin-coating technology have attracted much interest [17].

Meanwhile, photocatalysis is the most promising advanced oxidation process due to its ease of use and being environmentally friendly [18,19]. Heterogeneous photocatalysis efficiently degrades organic pollutants. However, some technical barriers limit the use of the photocatalyst: first, the wide bandgap of photocatalysts, which limits their usage to UV irradiation; second, inefficient integration into stationary reactors or limited recovery of unfixed photocatalysts after water treatment; third, low durability and efficiency of the photocatalytic treatment process [19].

Our previous study found that the combination of nanorods ZnO and rGO led to an increase in the photocatalytic activity of the photocatalyst. We successfully synthesized ZnO NRs hydrothermally in our earlier work, covered them with reduced graphene oxide, and investigated their optoelectronic, physical, and chemical properties [20,21]. GO-based nanomaterials can significantly increase the photocatalytic activity of ZnO because of their π-conjugated structure, which demonstrates excellent electronic mobility. This promotes the electron–hole pair separation on the ZnO surface and improves the harvesting of light energy in the visible range [22]. Previous reports also revealed that when carbon-based materials such as graphene and graphene oxide are added to photocatalysts, they may reduce the bandgap of the material and enhance the photocatalytic activity for the application of hydrogen production and contaminant degradation [23,24,25,26,27]. Moreover, the hydrothermal method is the most convenient method as it involve low temperatures with well crystallization to prepare ZnO based binary and multiple heterojunction [28].

In addition, microreactors provide several advantages for water purification, including a substantially shorter reaction time and uniform irradiation across the entire reaction surface area. Despite breakthroughs in material research and many seemingly promising configurations such as integrated photocatalysis–thermolysis water purification, microreactors’ low throughput remains a significant barrier to their use. These microreactors aid in overcoming issues with bulk reactors including photon transfer, mass transfer, inadequate oxygen supply, and uncontrolled reaction pathways. Miniaturized systems are advantageous, particularly for analytical applications, because they require only trace amounts of samples and reagents. This effectively reduces the time and effort needed for sampling and sample preparation while producing minimum waste [29,30].

There are several microfluidic reactor configurations with immobilized photocatalysts, which are micro-capillary, single microchannel, multi-microchannel, and planar. Micro-capillary has issues with difficult inner wall coating, and clogging may occur. A branched network of microchannels improves turbulence, increases residence time, and provides more area for light collection, while the planar design has more surface area [31].

Microfluidics and water purification appear incompatible because the former is designed to handle small amounts of the solution while the latter requires high throughput. This gap can be filled by increasing the size of the microreactors and using multiple microreactor parallel arrays. Alternatively, microreactors can be used for applications that do not require high throughput but repetitive tests, such as quick photocatalyst tests, operating condition optimization, and rapid screening of various photocatalysts [31].

According to earlier research, a contaminated water sample comprising a combination of benzene, toluene, ethylbenzene, and xylene can be degraded by a zinc oxide microfluidic reactor [32]. In this report, we show how the integration of rGO-deposited ZnO nanorods into a microfluidic reactor significantly reduces reaction time, which is reduced to a few seconds due to improved diffusion time, with the added ability to precisely adjust the water flow rate to better control the degradation efficiency. The latter is also intrinsically improved since the water sample flows readily through a small reactor with a high surface-to-volume ratio, enabling high throughput and fast water–photocatalyst interaction. The water treatment demonstration shown here is based on the use of methylene blue as a model chemical contaminant.

## 2. Synthesis and Fabrications Methods

### 2.1. Nanorods Synthesis and Characterization

The ZnO NRs were synthesized using the hydrothermal method and then deposited with rGO. Three steps were involved in the hydrothermal synthesis of the ZnO NRs. The first step includes the preparation of a precursor solution; zinc acetate dihydrate (0.01 M) and sodium hydroxide (0.09 M) were dissolved in methanol. Then, the glass substrate was spin-coated with the precursor solution and annealed at 150 °C for 10 min. The third step included growing ZnO NRs via the hydrothermal method at 95 °C. The growing solution was prepared by mixing equimolar aqueous solutions (0.025 M) of zinc nitrate and hexamethylenetetramine. All the mentioned chemicals were purchased from Chemiz (Shah Alam, Malaysia) and were used without further purification.

Next, the ZnO NRs/rGO were prepared by coating the ZnO NRs with GO and then annealed at 170 °C for 1 h. The deposition solution was prepared using GO (dispersion in water) obtained from iBiotool.com. GO dispersion in water (Biotool.com, Cambridge, UK) with a concentration of 2 mg/mL was diluted in DI-water to 0.075 mg/mL with flake sizes between 200 and 500 nm.

The synthesized nanorods were characterized in terms of their physical structure and chemical compositions. The surface morphology was analyzed using a Supra 55VP, Zeiss field emission electron microscope (FESEM; Zeiss, Oberkochen, Germany) at an accelerating voltage of 3 kV. While the sample’s crystal structure and growth direction were analyzed using a D8 Bruker Advance X-ray diffractometer with Cu ka radiation (λ = 1.54 nm) and X-ray diffraction (XRD)—Bruker (Billerica, MA, USA). The average crystallite size was obtained from the X-ray line extended analysis using the Debye–Scherrer method [33,34]. The detailed material properties of the ZnO NRs/rGO-based nanorods have been reported in our previous report [18].

### 2.2. Design of the Microfluidic Device

**Figure 1:**
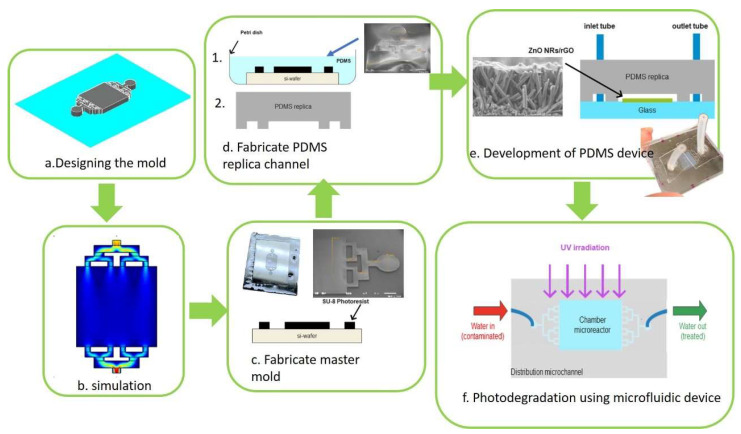
The microfluidic device fabrication steps: (**a**) The 3D mold design. (**b**) Numerical fluid flow simulation. (**c**) Fabrication of master mold (SEM image of SU-8 master mold). (**d**) PDMS casting for microchannel (SEM image of PDMS microchannel). (**e**) The microchannel and PDMS layers are bonded together using plasma bonding to fabricate the microfluidic device (cross-section view of the ZnO NRs/rGO nanocomposite). (**f**) Photodegradation setup, reprinted from Ref. [32].

The microfluidic device contains three main parts: the microchannel, reaction chamber, inlet, and outlet shown in Figure 1a. The height depth of the microchannels is 115 µm. The microchannel design is tree-branched which is aimed to produce a lower pressure drop in a fluid flow system caused by the resistance flow, resulting in longer residence time [35]. The design of the microfluidic structure and geometry was done using AutoCAD 2022 (Autodesk Inc., San Rafael, CA, USA) which is required for the master mold fabrication.

In many microreactors, the tree-branched bifurcating flow distributor is frequently utilized [32,36]. The advantages of this distributor include uniform water fill and low-pressure drop [37]. The tree-branched bifurcating, however, calls for a larger channel by tiers. The channel width was reduced by a ratio of 1.6 in this design, progressively reducing the flow. Assumptions: The models were created using the following presumptions: small particles in the water were ignored, any heat exchange was disregarded, and the incoming water flow rate was assumed to be constant.

A series of finite element calculations (FEM) was carried out in COMSOL Multiphysics Version 5.2 (COMSOL AB, Stockholm, Sweden) to study the velocity and pressure drop distribution inside the microfluidic device. The study was conducted to find the optimum flow rate range for the photocatalytic process.

Flow distribution analysis yielded the magnitude of pressure drop and flow velocity. The equations for calculating flow velocity and pressure using the laminar flow interface of the COMSOL Multiphysics Fluid Flow plug-in are the following equations, also known as the Navier–Stokes equations:

(1) $$\rho \frac{\partial U}{\partial t}+\rho \left(u.\nabla \right)u=\nabla .[-\mathrm{pl}+\mu (\nabla u+{\left(\nabla u\right)}^{T}-\frac{2}{3}\mu \left(\nabla .u\right)]+F$$

(2) $$\rho \nabla \;\cdot \;\left(u\right)=0$$

where *ρ* is the density, *u* is the flow velocity, *t* is time, *∇* is divergence, *p* is the pressure, −*pI* is the volumetric stress, and *F* is *pg* which is the body force.

During simulation, the Navier–Stokes equations are solved with no-slip boundary conditions to calculate the pressure and fluid velocity in the model. A time-dependent study is performed to show how the flow velocity and pressure change over time. In this simulation, we were given a water density *ρ* and an initial velocity *u*. The time was set to 1 second and the time step value was set to 0.001 s.

As shown in Figure 1b, COMSOL Multiphysics used the variation time t to calculate the flow velocity and pressure in each region of the mesh at each time step. All boundary conditions for all walls were set to non-slip. Water flows from above and out from below. Simulations were performed for the microreactor at various initial flow rates of 100 μL/min, 250 μL/min, and 500 μL/min.

### 2.3. Fabrication of the Microfluidic Device

The microfluidic device was made of PDMS (polydimethylsiloxane) due to its affordability compared to silicone, ease of fabrication, and optical transparency. The device was fabricated using soft lithography, which is an extensive and reliable method for creating polymer-based microfluidic devices [38]. The device consisted of three main parts, namely, a microchannel, reaction chamber with three branches, an inlet, and outlet.

The fabrication process of the microfluidic device started with photolithography to form a SU-8 based master mold using SU-8, as shown in Figure 1c. The process was followed by soft lithography to produce a replica of the PDMS in the SU-8 master mold, as shown in Figure 1d. A PDMS replica is shown in Figure 1e. The inlet and outlet were fabricated together during molding to create a fluid passage, as shown in Figure 1f. Then microfluidic integration was performed to attach the PDMS layer to the glass substrate coated with the ZnO NRs/rGO photocatalyst using corona discharge. The upper part is the PDMS layer which contains a microstructure consisting of microchannels, a reactor chamber, and inlet, and outlet fluid channels. The second part is a layer of glass coated with the ZnO NRs/rGO photocatalyst in the middle part, which is in the reactor chamber part.

Figure 1f shows the cross-section of the microstructure design. The fabricated microchannel has a high aspect ratio with a height and depth of 100 µm and 115 µm, respectively. The area of the reactor chamber is approximately 0.6 × 0.6 cm^2^. The inlet and outlet have a tube diameter of 1 mm. The geometry of the microreactor chamber with the tree-branched bifurcating distribution design model is shown in Figure 1f, and the variable parameters are displayed in Table 1. A scanning electron microscope (JEOL USA, Peabody, MA, USA) was used to evaluate the geometry of the master mold, as shown in Figure 1c, which was used for PDMS casting, as shown in Figure 1d.

### 2.4. Photodegradation Test Setup

**Figure 2:**
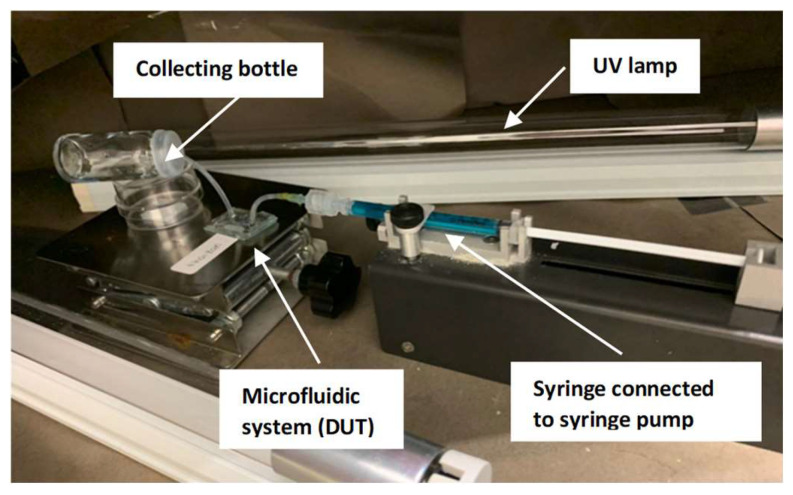
Photocatalytic experiment setup.

Studies on photocatalysis were performed on a manufactured nano-enabled microfluidic reactor. Two UVC lamps (18 W, λ = 254 nm (2.7 W/m^2^)) were used to illuminate the reactor. The distance between the UV lamp and the sample was 10 cm. An electrically monitored syringe pump (TS-2A Syringe Pump Controller, LongerPump, Longer Precision Pump Co., Halma Plc, Amersham, UK) was used to precisely manage the amount of water flowing through the microreactor chamber at a rate of 100, 250, 500, 750, and 1000 µL/min. Due to the limitation of our instrument, 100 µL/min is the minimum range for the flow rate. Using a Hitachi U-3900H double beam UV-Vis spectrometer (Hitachi High-Technologies Corporation, Tokyo, Japan), 3 mL of the water samples was examined both before and after treatment. A 0.05 mM solution of methylene blue (Sigma-Aldrich, St. Louis, MO, USA; 0.5 g/100 mL) was prepared with the initial pH of 6.86 as a sample of contaminated water. A methylene blue molecule measures approximately 9.5 Å in width and 13.82 Å or 14.47 Å in length [39]. The setup is shown in Figure 2.

(3) $$R=\frac{C_{o}-C_{t}}{C_{o}}\times 100\%\;$$

where C_o_ is the initial MB solution concentration and C_t_ is the MB solution concentration after treatment.

## 3. Results and Discussion

### 3.1. Morphology of the Nanocomposite Photocatalyst

**Figure 3:**
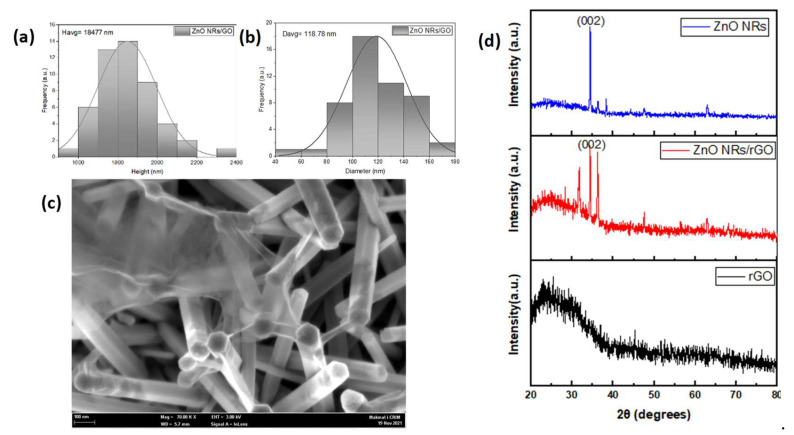
SEM image of the ZnO NRs/rGO (**a**) height distribution, (**b**) diameter distribution, (**c**) morphology, and (**d**) XRD spectrum of ZnO, ZnO NRs/rGO, and rGO.

The height and diameter distribution of the synthesized ZnO NRs/rGO is shown in Figure 3a,b, respectively. The average height and diameter of the ZnO NRs coated with rGO are 1847.77 nm and 118.78 nm. Figure 3c shows a ZnO NRs/rGO morphology on a glass substrate that was integrated inside the microreactor chamber. In Table 2, the edx results reveal that the presence of carbon in GO-coated ZnO NRs indicates a successful coating. The wrinkled layers of rGO are covered on top of the ZnO NRs. The rGO layers will facilitate the effective transport of excited electrons from the ZnO conduction band to the rGO surface due to their conductivity and capacity to suppress electron–hole pair recombination, boosting the photocatalytic activity of the nanocomposite. Figure 3d shows XRD patterns of ZnO NRs, rGO, and ZnO NRs/rGO. ZnO NRs and ZnO NRs/rGO show a major peak that could be assigned to ZnO (002), indicating that ZnO NRs and ZnO NRs/rGO present a hexagonal wurtzite structure with a (002) preferred orientation given at planes 31.8°, 34.4°, 36.3°, 47.5°, 56.6°, 62.9, 66.4°, 68.0°, 69.1°, 72.6°, and 77°. As shown, rGO has a dominant and broad diffraction peak centered approximately at 2θ = 24.5° [40]. They are also observed in the XRD pattern of ZnO NRs/rGO, which indicates that the nanocomposite has been successfully synthesized. In Table 3, the crystallite size of the ZnO NRs and ZnO NRs/rGO photocatalysts are the same as the GO coating does not affect the structure of the pre-synthesized ZnO NRs. The Debye–Scherer equation is used to determine the average crystallite size (D) of the sample using the full width at half maximum (FWHM = β) [41]. In our previous work, the microstructural properties of ZnO NRs/rGO have been investigated by XRD, UV- Visible (UV-Vis), and PL spectroscopies [21].

(4) $$D=\frac{0.94\;\lambda }{\beta \;\mathrm{Cos}\;\theta }.$$

### 3.2. Simulation Data

**Figure 4:**
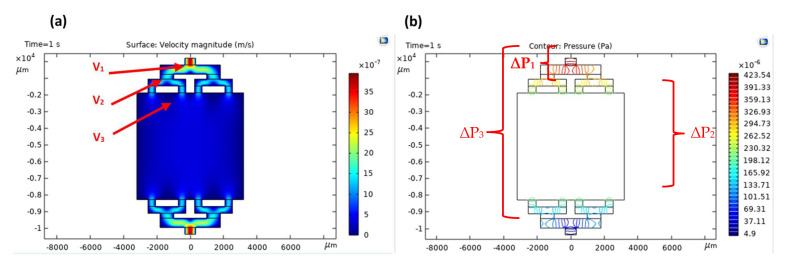
Velocity (**a**) and pressure (**b**) magnitude plot of the tree-branched bifurcating distributor model at a flow rate of 100 µL/min.

Figure 4a,b show the velocity magnitude and pressure of the tree-branched bifurcating distributor model of the microfluidic device. The graph shows the results at the last moment of the simulation, which is 1 second. Figure 4a shows the velocity variables with a legend representing the velocity in m/s. Figure 4b shows the pressure with a legend expressing the pressure in Pa. The flow velocity gradually decreases when water flows through the distributor channel. It also can be analyzed that the pressure gradually decreases as the water flows from the inlet to the outlet.

The parameters obtained from the result of the simulation are as follows: V_1_ is the maximum flow velocity at the inlet. V_2_ is the maximum velocity at the second-tier flow distributor channel. V_3_ is the approximate average flow velocity in the reactor chamber. ΔP_1_ is the pressure drop between the inlet channel and the second-tier distributor channel. ΔP_2_ is the pressure drop between the inlet and outlet ports. ΔP_3_ is the pressure drop between the upper and lower second-tier distributor channel connected by the reactor chamber. The approximate residence time R_t_ of the solution in the reaction chamber can be calculated using V_3_ and the area of the reaction chamber. The data are shown in Table 4. Flow rate Q (for rectangular channel) and effective residence time R_t_ were calculated using the following formulas:

(5) Q=v A

where v is the fluid velocity, and A is the cross-section area of the microfluidic flow.

(6) $$\;\mathrm{Effective}\;\mathrm{residence}\;\mathrm{time},{\;R}_{t}=\frac{\mathrm{Chamber}\;\mathrm{volume}}{\mathrm{Flow}\;\mathrm{rate}}$$

Based on the simulation study, as the initial flow rate is low, the lower pressure drop results in a longer residence time, thus an increase in photocatalytic activity. The pressure drop in the fluid flow system depends on the resistance of the flow. Thus, the ideal initial flow rate for this microreactor chamber design is 100 µL/min, resulting in more time for photocatalytic activity to take place. The results obtained are supported by experimental results, which at the lowest initial flow rate, 100 µL/min, the highest degradation of MB is achieved (Refer to Section 3.3).

### 3.3. Dye Photodegradation Experiments

Methylene blue (MB) has been used as the reference (model) contaminant in this experiment [31]. By appropriately diluting the MB solution in water, a calibrated solution of 0.05 mM was prepared. A 1 mL syringe was connected to a syringe pump and attached to the chamber system. The MB solution flows through the branched channel inside the PDMS chamber and is controlled using a syringe pump. The photodegradation tests were then done by exposing the flowing samples using two 18-Watt UV lamps. The outlet’s degraded MB water solutions were collected and subjected to spectroscopic analysis.

Our investigation began with the analysis of the UV-exposed fluidic samples using UV absorption spectroscopy. We compared the contents of the 3 mL initial sample solution with the treated sample solution after one pass through the microchamber integrated with the ZnO NRs/rGO photocatalyst.

**Figure 5:**
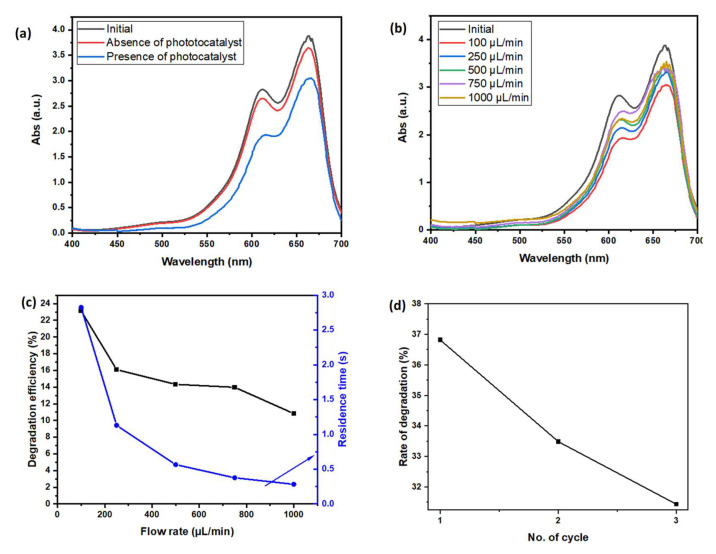
UV absorption spectroscopy of MB solution before and after one-pass photocatalytic degradation within the microreactor at a flow rate of 100 µL/min (**a**), spectral changes during degradation of MB before and after a one-pass photocatalytic degradation within the microreactor containing ZnO NRs/rGO at varying initial flow rates (**b**), the degradation rate of MB solution before and after a one-pass photocatalytic degradation within the microreactor containing ZnO NRs/rGO at varying initial flow rates (**c**), and repeatability test results (**d**).

Utilizing a reactor containing ZnO NRs/rGO reduces absorption substantially. Figure 5a shows the two absorbance spectra before and after one-pass degradation under an optimal flow rate (100 µL/min). As demonstrated in Figure 5b, the difference between before and after the photodegradation process with the presence of ZnO NRs/rGO is more visible in the spectral region 613 to 664 nm. A comparable microfluidic sample without ZnO NRs/rGO was used for a control experiment, with only a slight reduction of absorption spectrum with the initial polluted water sample. We can conclude that the ZnO NRs/rGO is responsible for the dye photodegradation process.

Figure 5b shows the UV absorption spectroscopy of the MB solution before and after a one-pass photodegradation within the microreactor containing the ZnO NRs/rGO at varying initial flow rates. The investigation of the effect of flow rate on the degradation rate was studied for various flow rates of 100, 250, 500, 750, and 1000 µL/min. The corresponding effective residence times are approximately 2.8, 1.1, 0.6, 0.4, and 0.3 s, respectively. The lower the initial flow rate, the longer the residence time as calculated using Equation (6). The longer the residence time, the more time for photoreaction to occur. Thus, a higher degradation efficiency of MB is achieved. The results of degradation efficiency at varying initial flow rates can be referred to in Table 5. The ideal flow rate used was 100 µL/min with an effective residence of 2.83 s. The pH of the initial MB was 6.86, and after the photocatalytic degradation, the pH was reduced to 6.64. The degradation reached an average of 23.12%. The ZnO NRs/rGO on this microfluidic platform was close to the water flow inside the microfluidic chamber and had a high surface-to-volume ratio.

The height of the ZnO NRs was 1400 nm but with rGO was 1847.77 nm, and its lateral dimension of the reaction chamber was approximately 6.4 × 6.4 mm^2^, a lateral dimension of the photocatalytic material substrate was 4 × 4 mm^2^, corresponding to a volume chamber of 4.7082 µL. To increase the degradation efficiency, the reactor size needs to be bigger.

From Figure 5c, the lower the solution flow rate flows into the inlet, the higher the residence time, thus more photocatalytic activity can occur, increasing the degradation efficiency inside the reactor chamber. Subsequently, the experimental results were compared with COMSOL simulation. The experimental results obtained are in good agreement with the simulation data, where the lower the initial flow rate, the lower the pressure drop and the velocity distribution inside the reactor chamber; thus, this increases the effective residence time for the photocatalytic activity to take place.

To study the stability of the device performance, the degradation of MB using a ZnO NRs/rGO nanocomposite-based microfluidic device was repeated three times. As shown in Figure 5d, the stability slightly decreases with an increasing number of cycles. The ZnO NRs are stable over time in an aqueous solution and degradation efficiency is shown to be stable.

**Figure 6:**
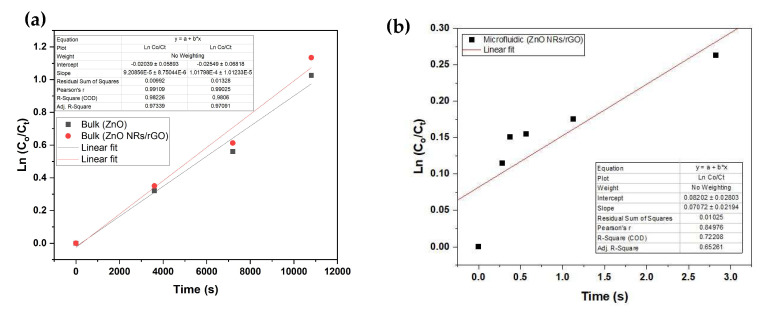
Correlation of ln (C_0_/Ct) and reaction time (**a**) bulk system and (**b**) microfluidic system.

The MB degradation kinetics using catalysts integrated into the microfluidic system is shown in Figure 6b and in the bulk system in Figure 6a. The graph demonstrates strong linearity between ln (C_o_/C_t_) and time, with a regression coefficient R^2^ close to 1 (Table 6). The first-order kinetic model accurately describes MB degradation under UV light. The reaction rate constant of photocatalysts in a microfluidic platform is higher than photocatalysts in a bulk system. The results show that under the same UV irradiation, the reaction rate of photocatalyst in a microfluidic system was 0.0707 s^−1^ which is almost 700 times higher than that in a bulk system. It is therefore proven that the microfluidic platform successfully enhanced the reaction rate of dye degradation.

Table 7 provides a comparison of the contaminant degradation process done in a microfluidic system. Our microreactor possessed a simple and low-cost fabrication process and acceptable photocatalytic activity using zinc oxide nanorods coated with reduced graphene oxide. The result shows that the photocatalyst process in a PDMS based microfluidic system obtains a higher degradation efficiency rate compared to that in a bulk system.

**Table 7:** Comparison of recent studies of photocatalytic degradation in microfluidic systems with our work.

Photocatalyst	Experimental Setup	Initial Flow Rate (µL/min)	Chamber Volume (µL)	Effective Residence Time (s)	Reaction Rate of Photodegradation	Reference
Film of anatase titania nanoparticle	UV 0.1 mM MB	12	2.97	14.85	10.6%/s	[42]
Flm of BiVO_4_	Blue Light LED NaCl: 0.1 mol/l	75	10	8	10.4%/s	[43]
**Nanorods composite of ZnO/rGO**	**UV** **0.05 mM MB**	**100–1000**	**4.7082**	**2.82**	**16%/s**	**Our work**

## 4. Conclusions

This study presented a nano-enabled photocatalytic microreactor for water purification by combining hydrothermally produced ZnO NRs/rGO on a glass substrate within a PDMS-based microfluidic chamber (6.4 × 6.4 mm^2^ × 80 µm). The purification of MB dye-contaminated water was evaluated using optical spectroscopy techniques. The suggested microreactor was demonstrated to be exceedingly efficient and quick as acceptable degradation efficiency was accomplished within an effective residence time of less than 3 s due to the large surface-to-volume ratio of the rGO-deposited ZnO nanorods and their close proximity to the water flow in the microfluidic chamber. The multiple microreactor parallel array is a potential way to increase throughput capacity, making the device more efficient and realistic for low-volume water purification.

## Figures and Tables

**Table 1:** Parameters of the microfluidic device.

Parameter	Width (µm)	Length (µm)
First channel	655.36	409.6
Second channel	3605.6	563.2
Third channel	819.2	292.8
Fourth channel	2265.6	413.6
Fifth channel	456	409.6
Reactor chamber	6392	6405

**Table 2:** Quantitative analysis of EDX.

Component	Atomic Weight Percentage %	
	ZnO NRs	ZnO NRs/rGO
Zn	79.9	71.7
O	20.1	21.5
C	-	6.9

**Table 3:** XRD analysis.

Materials	2θ(101)	FWHM	D (nm)	c (Å)
ZnO NRs	36.22	0.189	46.21	5.2066
ZnO NRs/rGO	36.30	0.189	46.21	5.2066

**Table 4:** Parameters comparison at varying initial flow rates.

Flow Rate (µL/min)	100	250	500
V_1_ (m/s)	36 × 10^−7^	10 × 10^−6^	19 × 10^−6^
V_2_ (m/s)	15 × 10^−7^	4 × 10^−6^	8 × 10^−6^
V_3_ (m/s)	5 × 10^−7^	2 × 10^−6^	4 × 10^−6^
P_1_ (Pa)	193.22 × 10^−6^	48.25 × 10^−5^	96.37 × 10^−5^
P_2_ (Pa)	386.42 × 10^−6^	96.49 × 10^−5^	208.81 × 10^−5^
P_3_ (Pa)	32.2 × 10^−6^	8.04 × 10^−5^	16.07 × 10^−5^
R_t_ (sec)	2.8249	1.13	0.5650

**Table 5:** Degradation of MB using the microfluidic device under light irradiation.

Flow Rate (µL/min)	Residence Time (s)	Degradation Efficiency (%)
100	2.8249	23.12
250	1.13	16.10
500	0.565	14.33
750	0.3767	14.00
1000	0.2825	10.83

**Table 6:** First-order kinetic equation and correlation coefficient R^2^ of the MB degradation reaction of the catalyst.

Material	The First-Order Kinetic Equation	Regression Coefficient, R^2^	Reaction Rate (Ka) (s^−1^)
ZnO NRs (bulk system)	y = 0.00009x − 0.0204	0.9823	0.00009
ZnO NRs/rGO (bulk system)	y = 0.0001x − 0.0255	0.9806	0.0001
ZnO NRs/rGO (microfluidic)	y = 0.0707x − 0.082	0.7221	0.0707

## Data Availability

The data presented in this study are available on request from the corresponding author.
